# Novel algorithm for a smartphone-based 6-minute walk test application: algorithm, application development, and evaluation

**DOI:** 10.1186/s12984-015-0013-9

**Published:** 2015-02-20

**Authors:** Nicole A Capela, Edward D Lemaire, Natalie Baddour

**Affiliations:** Ottawa Hospital Research Institute, Ottawa, Canada; Faculty of Medicine, University of Ottawa, Ottawa, Canada; Mechanical Engineering, University of Ottawa, Ottawa, Canada

**Keywords:** Rehabilitation, Gait, Mobile computing, Smartphone, Accelerometers, Body-worn sensors, Inertial measurement unit, Application software, Software design

## Abstract

**Background:**

The 6-minute walk test (6MWT: the maximum distance walked in 6 minutes) is used by rehabilitation professionals as a measure of exercise capacity. Today’s smartphones contain hardware that can be used for wearable sensor applications and mobile data analysis. A smartphone application can run the 6MWT and provide typically unavailable biomechanical information about how the person moves during the test.

**Methods:**

A new algorithm for a calibration-free 6MWT smartphone application was developed that uses the test’s inherent conditions and smartphone accelerometer-gyroscope data to report the total distance walked, step timing, gait symmetry, and walking changes over time. This information is not available with a standard 6MWT and could help with clinical decision-making.

The 6MWT application was evaluated with 15 able-bodied participants. A BlackBerry Z10 smartphone was worn on a belt at the mid lower back. Audio from the phone instructed the person to start and stop walking. Digital video was independently recorded during the trial as a gold-standard comparator.

**Results:**

The average difference between smartphone and gold standard foot strike timing was 0.014 ± 0.015 s. The total distance calculated by the application was within 1 m of the measured distance for all but one participant, which was more accurate than other smartphone-based studies.

**Conclusions:**

These results demonstrated that clinically relevant 6MWT results can be achieved with typical smartphone hardware and a novel algorithm.

**Electronic supplementary material:**

The online version of this article (doi:10.1186/s12984-015-0013-9) contains supplementary material, which is available to authorized users.

## Background

In a healthcare environment, exercise capacity measurement is important for understanding a person’s current status and evaluating rehabilitation improvement. The 6 minute walk test (6MWT), where the distance walked in 6 minutes is measured, is a common clinical tool for this purpose. A smartphone with integrated sensors provides a viable platform for wearable biomechanical applications. For the 6MWT, wearable analysis can derive additional information with minimal additional setup, providing clinically useful and immediate output for evaluating physical function and gait characteristics at the point of patient contact, without the need to purchase specialized medical equipment.

Wearable sensors allow a person to walk freely, at a self-selected and natural pace that is more representative of daily living than some laboratory conditions [1]. Many studies have used accelerometers for gait detection and to compute gait parameters such as cadence, step timing, and symmetry [1-3] (Table 1). Accelerometers can also be used to measure physical activity levels that correlate with 6MWT results [4]. The current paper presents a novel algorithm that utilizes the 6MWT’s unique constraints and multiple sensors that are readily available in smartphone platforms to calculate clinically useful 6MWT outcomes.

**Table 1:** **Summary of recent accelerometer-based step counting studies**

**Study**	**Walking conditions**	**Sensor location**	**Equipment (sampling rate)**	**Step detection algorithm**	**Goal**	**Results**
Ying (2007) [20]	Treadmill	Lateral side of left and right foot	Dual axis accelerometers (200 Hz)	Pan Tompkins, template, dual axis peak detection	Accurate step detection	Qualitative comparison
Zijlstra (2003) [24]	Hallway	Trunk	Triaxial accelerometer (100 Hz)	Peaks preceding sign change in forward acceleration	Foot strike	Within 0.02 s (SD <0.03)
Huang (2012) [10]	Treadmill	5 locations	HTC smartphone (10 Hz)	Threshold from training period	Count steps	93-96% step count Accuracy
Naqvi (2012) [11]	Level ground	Near centre of mass (COM)	Smartphone (100 Hz)	Adaptable threshold	Count steps	1-2 step error (of 15-40 steps)
Kim (2004) [22]	Hallway	Ankle	MEMS accelerometer, vertical and forward (100 Hz)	Sequential thresholds to recognize swing phase, foot strike	Count steps, estimate distance	<1% step count error 5% distance error
Yang (2012) [3]	25 m, hallway	Lower back in belt	HTC smartphone (25 Hz)	Peaks preceding sign change in forward acceleration, manually verified	Foot strike, regularity, symmetry	Visually verified to 100% accuracy
Ayub (2012) [12]	Hallway	3 locations	HTC smartphone (25 Hz) interpolated 50 Hz	Zero crossing and threshold lengths, Variance detector	Step count, stride length	1.5-5% step count error
Derawi (2010) [23]	20 m, level ground	Left leg by hip	Accelerometer (100 Hz)	Neighborhood search for minimum peaks	Cycle detection, distance metric	EER = 5.7%
Martin (2011) [25]	Varying speeds	Varying locations	Accelerometer (30 Hz)	Continuous wavelet transform (CWT)	Stride length (step counting)	Not reported
Kim (2013) [21]	Treadmill varying speeds	Left waist	Triaxial accelerometer (32 Hz)	Heuristic, adaptive threshold, adaptive locking period	Step count and activity monitoring	97% Recognition rate

Commercial products are available that count steps, such as accelerometer-based devices for activity and sleep monitoring purposes like the Actigraph activity monitor [5] and StepWatch 3 Activity Monitor (SAM) [6]. Actigraph counts steps with an error rate of <1% at normal speeds and approximately 5% at slow speed (0.83 m/s) [5]. SAM uses a dual axis accelerometer and microprocessor, worn on the ankle, with a step counting accuracy of 98-99% [6].

Other commercial products are designed specifically for medical use. The Actibelt® incorporates a 3D accelerometer into a belt buckle to record accelerations close to the body’s centre of mass [7]. Several clinical tests have been programmed for the Actibelt®, including the 6MWT. The Aipermon Medlog 200 also offers a “start 6MWT” button and automatic recording termination after 6 minutes [8,9]. These products were specifically designed for medical purposes and require the purchase of, and familiarization with, specialized commercial equipment and software. A 6MWT smartphone application (app) would provide an affordable and accessible means of obtaining the same information in the person’s home or by a healthcare provider at point of patient contact, without the need for additional data acquisition hardware. Smartphones also offer multiple additional sensors, such as gyroscopes, that can be used to improve 6MWT result accuracy.

Various studies have demonstrated the viability of smartphones for step counting or gait analysis, which makes them a feasible tool for automating the 6MWT [3,10-14]. Smartphone accelerometers typically have slower sampling rates that vary [15], in contrast with purpose built data collection systems that provide a fixed and reliable sampling rate. This adds additional signal processing requirements for valid human activity analysis.

Annegarn et al. used accelerometers to assess walking patterns during the 6MWT [16]. Steps were detected as the peak forward acceleration changed from positive to negative, identifying foot strike [17]. Annegarn et al. did not assess the algorithm’s accuracy but used the results to observe differences between healthy controls and patients with chronic obstructive pulmonary disease (COPD). The 6MWT was used as an accelerometer gait data source, with the first and last five seconds removed to exclude irregular walking patterns. Participants who stopped during the test were excluded. The algorithm did not run the test or estimate the total distance walked.

Cheng et al. developed a smartphone application (GaitTrack) that monitored walking patterns using accelerometers [18]. Juen et al. [19] used GaitTrack to run the 6MWT with 30 COPD patients. To our knowledge, this is the only other smartphone application reported in the literature to fully run and calculate outcomes for the 6MWT. This research demonstrated that smartphone sensors can detect steps with comparable accuracy to commercial medical pedometers. GaitTrack implemented “activity recognition” to differentiate between walking and non-walking activities, recording only during walking. A linear regression model was trained to estimate stride length, using eight parameters derived from the smartphone accelerometer. Stride length was multiplied by the counted steps to calculate distance walked. The model was trained and tested using 10-fold cross validation, which used the same data for training and testing. The average test distance error was 5.87%. Using their average total distance of 276.2 m, this percent error corresponded to a distance error of 16.2 m (i.e., longer than the 15 m trial walkway). This error could have been reduced by using other phone sensors to identify turns and differentiate them from walking or standing, rather than simply dividing the data into “walking” or “not walking” sections. In this paper, we report on an algorithm that uses the inherent 6MWT constraints and multiple smartphone sensors to count the number of walkways completed and provide a more accurate distance calculation.

The 6MWT is a simple test that requires minimal equipment and is implemented regularly to evaluate a person’s physical capacity. With the emergence of multiple sensors in smartphones, these wearable computing platforms can easily and quickly provide additional information on how the person moves during a test. However, the ability to provide clinically acceptable accuracy for 6MWT distance is needed first to make this a relevant tool (i.e., first prove that the 6MWT outcome is viable). This study developed and evaluated the performance of a custom 6MWT smartphone application developed to run the test, detect foot strikes, and calculate distance walked with no calibration required.

## Methods

### Algorithm and application development

**Figure 1:**
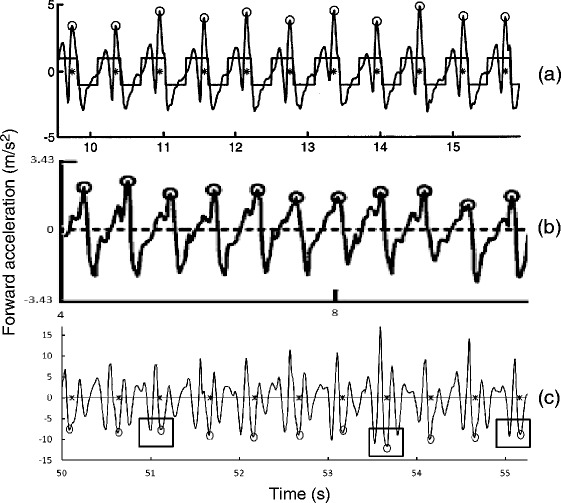
**Forward acceleration with circles identifying foot strikes. (a)** Triaxial accelerometer at 100Hz, filtered at 20Hz, with asterisks showing foot strike (modified from Zijstra [24]); **(b)** Android smartphone accelerometer downsampled to 50Hz (modified from Mellone [15]); **(c)** Representative data sample from the current study showing similar peaks, highlighted by squares, which would produce incorrect step identification without a locking period. Raw signal is inversed to match convention used by [24] and [15] and asterisks represent foot strike.

When walking, the acceleration signal’s cyclical nature permits step identification using the amplitude at each peak and the time between each peak or zero-crossing. Some basic algorithms set a minimum amplitude threshold that, when surpassed, identifies a step [10,11,20-22]. This approach can be problematic if acceleration fluctuates throughout the gait cycle, introducing false peaks. The time between steps is often used to set a “locking period” during which a second step is not expected [12,20,21,23]. The locking period method requires accurate peak or zero crossing identification. Marschollek et al. [2] compared healthy and mobility-impaired participants using four step counting algorithms: Pan-Tompkins, Dual-Axis, Wolf, autocorrelation. Algorithms that adapted to periodic acceleration patterns, rather than relying on a-priori knowledge of the gait signals, were more adaptable to mobility-impaired participants. Marschollek recommended more complex pattern classification algorithms to recognize steps in samples with differing motion characteristics. Annotated sample forward acceleration signals obtained from our data set, as well as those of Zijlstra et al. [24] and Mellone et al. [15], are shown in Figure 1. In this figure, the acceleration signal from our data set was inversed to match the convention of Zijlstra and Mellone [15,24]. As outlined with square boxes, signals for some people produce similar consecutive peaks and zero crossings. Therefore, a combination of signal processing methods is required to reliably identify steps. The current work built on peak detection and locking period methods and implemented an adaptive signal shape recognition algorithm for reliable step recognition (Section “Processing and Algorithm”).

Distance traveled can be calculated by double integrating the acceleration; however, this requires careful calibration, extensive computation, and works best when the accelerometer is mounted low on the person’s body (i.e., on the foot) [25]. Distance may also be estimated by calculating the stride length using empirical relations with other measurements, including leg length, change in acceleration, and step frequency. The estimated stride length is then multiplied by the number of strides to determine the distance traveled, thereby relying on a consistent stride length. The empirical relationships derived in various studies, such as the Weinberg algorithm, rely on parameters that must be calibrated to each individual from experimental walking data [14]. This requires leg length measurement or participant height for inversed pendulum models [12,26], or determination of constants from walking trials [22,25]. Alternatively, mean step length can be reliably estimated when the distance walked is known [27]. The 6MWT is typically performed on a straight track of known length and therefore offers an ideal opportunity to estimate mean step length for use in total distance calculations.

#### Processing and algorithm

##### Turns

Since a person walks back and forth on a predetermined straight track during the 6MWT, turns at the end of the straight track must be identified to accurately divide the data into lengths. These lengths were analyzed separately and steps during turns were not counted, since they did not contribute to the distance walked and would alter the interpretation of linear walking gait characteristics.

**Figure 2:**
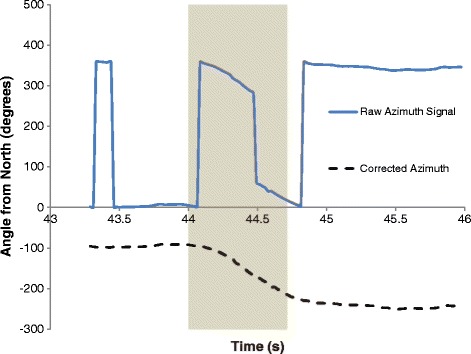
**Raw and corrected azimuth signals (turn highlighted).**

**Figure 3:**
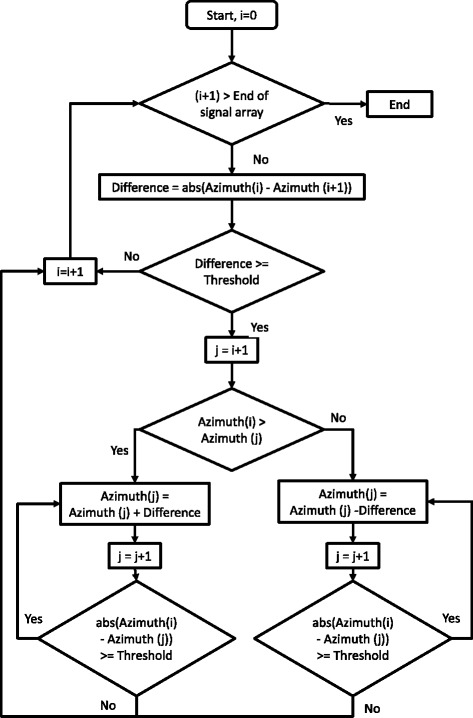
**Azimuth correction flowchart.** The threshold was 10°.

Turns were identified using the azimuth signal output from the BlackBerry Z10 smartphone, which was derived from the gyroscope and magnetometer sensors. For this application, azimuth was the angle between the device axis normal to screen and magnetic north. This signal stayed between 0 and 360°, which caused rapid changes in magnitude (Figure 2). This was corrected in software before detecting turns. If a 10° difference between data points was found, the difference was added or subtracted from the signal to correct the curve without losing information on genuine signal changes. The signal correction algorithm is shown in Figure 3.

A turn was detected if azimuth changed by more than 100° within a 3 second window. For each detected turn, turn duration was defined as the azimuth signal section with a standard deviation greater than 10° per 1 second timeframe. These were reliable ranges for turn detection at different speeds.

##### Step detection and step timing

Steps were detected using the BlackBerry Z10 linear acceleration signal (i.e., device acceleration minus acceleration due to gravity). This signal was filtered using a fourth-order zero-lag Butterworth low pass filter with a 4Hz cutoff frequency. A low cutoff frequency was acceptable since the filtered signal was only used to detect step occurrence and the raw forward acceleration signal was used to identify foot strike time. Cutoff frequencies lower than 4 Hz resulted in missed steps.

A step detection method was developed using a combination of adaptive locking period, similar to [21], peak detection, as in [3,17,28], and a custom adaptive signal shape template. First, the locking period was calculated using a 5 second sample from the filtered vertical acceleration signal at the beginning of the 6MWT trial, therefore no extra training data were required and the locking period was specific to the individual’s results. To establish the locking period, the time was calculated between consecutive positive zero crossings of the vertical acceleration signal. This signal did not cross zero on every step, since the filtered vertical linear acceleration signal sometimes fluctuated from a zero baseline (i.e., the time between zero crossings did not necessarily reflect step duration). Therefore, additional procedures were used to calculate the locking period:

- As default, the locking period was half the maximum time between zero crossings.
- If the maximum time between zero crossings was greater than a preset threshold (0.7 s, which is longer than typical step time), the locking period was half the mean time between zero crossings.
- If the maximum time between zero crossings was less than another threshold (0.4 s, which is shorter than typical step time), the maximum time between zero crossings was multiplied by 0.6.

The number of changes in direction (positive or negative peaks) in the 5 second sample was counted for both forward and vertical acceleration signals. The filtered forward acceleration signal was the default for step detection; however, if the ratio of forward and vertical direction change-counts was greater than 1.4, vertical acceleration was used to detect steps. This 1.4 ratio was determined by observation from a separate data set. In this way, the more appropriate signal for step detection was selected, based on the individual’s walking style.

The 5 second calibration sample was also used to calculate individual thresholds to detect the first and last steps in a walkway, because these steps tended to have lower peak values since the person was starting up from, or slowing down for, a stop or turn. These thresholds were calculated by subtracting the mean filtered forward acceleration from the maximum in the 5 second sample.

**Figure 4:**
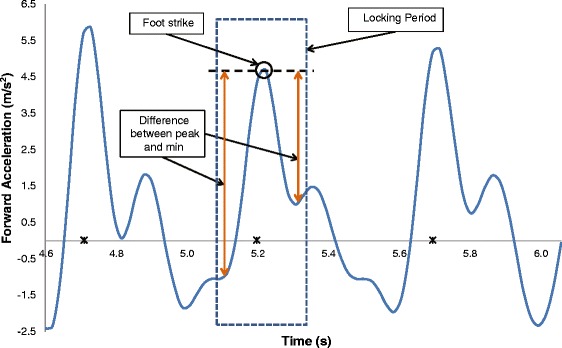
**Step detection.** The dashed square represents the locking period, the circle is the detected peak and the arrows indicate the difference between the peak and min on either side. Asterisks represent foot strikes.

The chosen filtered acceleration signal was searched using a moving window with the size of the locking period, shown as a dashed square in Figure 4 (foot strike was detected from the video recording). In each window, the maximum peak acceleration was used to detect one step, shown as a circle in Figure 4. Different people produced different peak amplitudes, with these peak segments being sharp and short, rounded and longer, or asymmetrical, depending on the person’s gait pattern. Thus, step identification was based on signal shape similarity to other identified steps in the same walk, rather than pre-assigned thresholds. Within the search window, differences between a peak and the minima before and after the peak (Figure 4) were calculated as:

1 $$ Lef{t}_{Diff}= \max \left( locking\  period\right)- \min \left( left\  side\  of\  peak\right) $$

2 $$ Righ{t}_{Diff}= \max \left( locking\  period\right)- \min \left( right\  side\  of\  peak\right) $$

A peak was only identified as a step if the difference between the peak and the minimum on either side were both within 35% of the calculated values from the previous step. This method was reliable when the signal drifted above or below a zero baseline.

3 $$ \frac{\ Lef{t}_{Diff}\left( current\  step\right)}{\ Lef{t}_{Diff}\left( previous\  step\right)}>0.35 $$

4 $$ \frac{\  Righ{t}_{Diff}\left( current\  step\right)}{\  Righ{t}_{Diff}\left( previous\  step\right)}>0.35 $$

If the duration between 2 consecutive steps was greater than 1.75 times the previous step, this portion of the signal was reanalyzed to check for missed steps. For all peaks in a section, three tests were used to determine if a step occurred:

1. The difference between the peak and the minimum on either side was within 30%.
2. The peak matched the timing pattern of previous step.
3. The acceleration that was not used for step detection (vertical or forward) passed the initial thresholds for the first and last steps.

**Figure 5:**
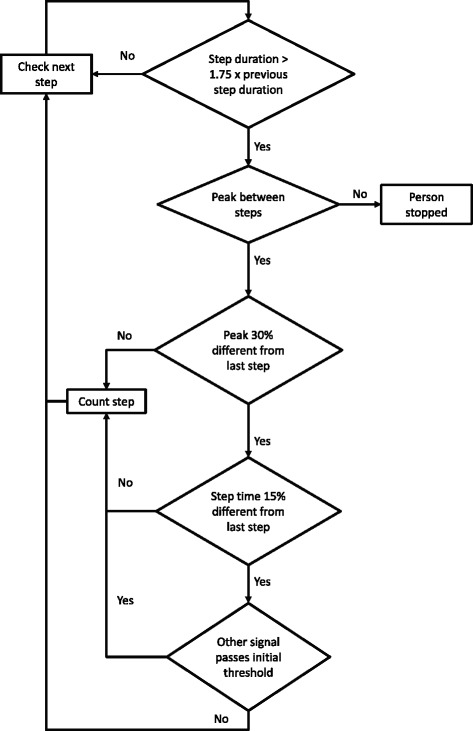
**Flowchart for detecting missed steps.**

If no missed steps were identified, or if the duration between identified steps and the next step was still greater than 1.75 times the previous step, this pause in steps was considered a stop (i.e., person stopped walking during the test). This process is depicted in Figure 5.

Once step occurrence was detected, foot strike time was determined from the maximum peak in the raw forward linear acceleration, within the neighborhood of each detected step. Since the phone faced backward, peak forward acceleration corresponded to the maximum negative acceleration during foot strike. This was typically the most prominent signal characteristic for foot strike identification.

##### Left and right steps

**Figure 6:**
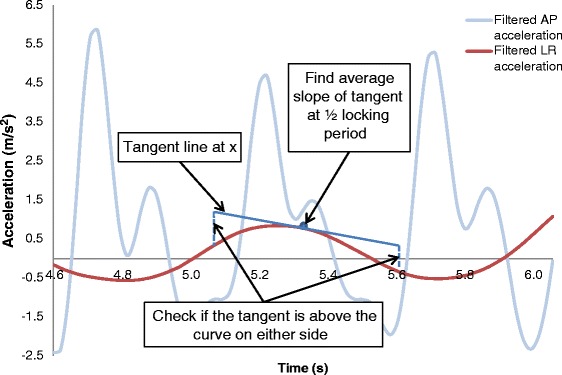
**Tangent method for identifying left and right steps.**

Left and right steps were identified using the left-right (LR) linear acceleration. This signal was filtered using a fourth-order zero-lag Butterworth low pass filter with 1Hz cutoff frequency. At each detected step, the tangent to the filtered LR linear acceleration signal was calculated at 0.25 of step duration, (*a*), using the following equation.

5 $$ y=\frac{\left(L{R}_{a+1}-L{R}_{a-1}\right)}{2}\left(x-a\right)+b $$

where *x* and *y* are the horizontal and vertical coordinates in the equation, respectively, of the tangent line; *a* is the *x*-coordinate of the LR acceleration at 0.25 step duration; and *b* is the *y*-coordinate at 0.25 step duration. *LR*_*a+1*_ and *LR*_*a-1*_ are the values of the filtered LR acceleration at times *a* + 1 and time *a*-1, respectively, which are used to find the average slope of the curve at *a*. If the *y*-value was greater than the filtered LR linear acceleration value at times (*a-locking period*) and (*a + locking period*), the person was accelerating to the right. *y*-values equal to or less than these values indicated acceleration to the left (Figure 6). A sequence of left and right steps were identified and used to fill in the steps that were not identified using the tangent method, as well as to correct double counts. This information was used to calculate the primary outcome measures.

##### Distance walked

The 6MWT consists of a person walking back and forth along a straight walkway of known length, as per American Thoracic Society guidelines [29]. This constraint provides the opportunity to calculate the distance without the use of additional measurements. Since a 6MWT can be performed on a walkway of any length, depending on the space available, the user must enter their walkway length before the test.

Once turns at the end of the straight portion of the walkway have been identified and steps counted, the number of steps for a predetermined distance (walkway length) is known and the mean step length can be found for each walkway. The distance walked on the last walkway was calculated using the number of steps multiplied by the average step length of the previous walkway. If a stop was detected in the previous walkway, the walkway with no stops, prior to the walkway when the stop occurred, was used to calculate average step length. If the ratio of mean step duration in the last walkway to step duration in the walkway before was less than 0.9 (i.e., more than 10% increase in the mean time of each step), the average step length was multiplied by this ratio before calculating the total distance.

The total distance walked is calculated as the number of full walkways completed multiplied by the known walkway length, in addition to the distance walked in the last (partial) walkway. With this approach, the step length error is not compounded across all walkway lengths. Since a delay was anticipated between the tone that signals the end of data collection and the person stopping, video was used to verify the actual distance traveled by the person in 6 minutes (i.e., video was used to identify the body location at 6 minutes and then direct measurement of the distance to this location).

### Evaluation

#### Participants

A convenience sample of 15 able-bodied staff and students was recruited from The Ottawa Hospital Rehabilitation Centre (TOHRC). Able-bodied participants are a viable proxy for patients with chronic obstructive pulmonary disease, a population that commonly use the 6MWT, since previous research found no significant differences for walking distance, intensity, and cadence [16]. The sample consisted of 10 males (Age = 40.6 ± 15.9 years, Mass = 78.5 ± 12.1 kg, Height = 177.5 ± 5.8 cm) and 5 females (Age = 38.8 ± 9.7 years, Mass = 64.5 ± 10.5 kg, Height = 165.2 ± 4.8 cm). Written, informed consent was obtained from each person before starting data collection. The study was approved by the Ottawa Health Science Network Research Ethics Board.

#### Protocol

After completing the consent form, participant age, height, sex, weight, waist size, and leg length were recorded. Before testing, a belt was secured around the person’s waist, with a rear pocket at the centre of their lower back. The app was started on a BlackBerry Z10 smartphone and the 25 m track length was entered into a text box. The 6MWT was selected from a drop down menu, from the choice of 2 or 6 minutes tests. The 6 minute test was chosen for evaluation to ensure that the algorithm worked for the longer test, and because the 6MWT is more commonly used in clinical practice. The smartphone was placed upright in the rear pocket, facing outward (therefore backwards). Smartphone audio instructed the participant to begin walking and stop walking when they hear a tone. Participants walked back and forth along a 25 m section of a straight hallway, covering as much distance as possible in 6 minutes. Accelerometer, gyroscope, and magnetometer data were sampled on the Z10 at approximately 50Hz. Smartphones can have a variable sampling rate [15], and the Z10 sensor sample rate varied with a mean standard deviation of 3.84 Hz for all subjects. For every trial, the person was video recorded using a separate and independent BlackBerry 9900 smartphone. At the end of the test, the distance walked on the last length was measured with a measuring tape and recorded on a data sheet as a comparator.

#### Outcome measures

Foot strike timing, number of steps, turns, and contextual information were extracted from the digital video as a gold-standard comparator. The gold standard time was synchronized with smartphone output by the first identified foot strike event, thereby providing a recognizable accelerometer signal and video event. The 6MWT algorithm was initially developed as a custom Matlab program that was then converted into a BlackBerry 10 app that runs entirely on the phone. The algorithm can be written to run on any commercial smartphone with an accelerometer and gyroscope. The app calculates and saves outcome data as a comma separated value (CSV) file, as well as saving a raw sensor data text file if desired. The outcome data file includes information that would be useful to clinicians; such as, average step length, step time, cadence over time, and cadence per walkway. The app also displays the total distance walked on the phone upon test completion. Individual foot contact times, which are used to calculate the outcomes but are not useful to clinicians on their own, are not included in the output text file. Therefore, to facilitate the evaluation of foot contact identification, a custom Matlab program was used to import the raw data text file, calculate outcomes, and compare results with gold standard outcomes.

The following information was calculated from sensor data: total distance walked, total number of steps, number of steps per walkway length, cadence average (AVG) and standard deviation (SD), step time AVG and SD (left and right steps), stride time AVG and SD, step time symmetry (left and right steps).

## Results

The measured distance and the total distance calculated by the algorithm for each participant are shown in Table 2. Total distance calculated was within 1 m of the measured distance in all trials except for Participant 11 (2.1 m error). The average error in calculated distance was 0.12%, markedly better than the average error of 5.87% reported in a recent 6MWT algorithm for smartphones [19].

**Table 2:** **Distance walked**

**Participant**	**Total Distance (m)**		
	**Measured**	**Calculated**	**Difference**
1	554.50	554.55	0.05
2	511.29	511.36	0.07
3	457.28	456.94	0.34
4	673.17	674.04	0.87
5	493.83	493.18	0.65
6	536.70	535.94	0.76
7	601.10	601.61	0.51
8	542.00	541.18	0.82
9	667.00	666.07	0.93
10	486.14	485.29	0.85
11	552.10	550.00	2.10
12	468.90	468.18	0.72
13	503.66	503.13	0.54
14	542.68	541.94	0.74
15	553.00	552.94	0.06
Average	542.89	542.42	0.67
Standard deviation	63.86	64.08	0.50

The time difference between foot strikes detected by the algorithm and foot strikes identified in the video are summarized in Table 3, as well as the total steps counted. For all participants except participant 6, forward acceleration was selected for analysis. One step was not counted for participant 6 (99.85% accuracy), and 4 steps were not counted for participant 3 (99.38% accuracy). All other steps were counted with 100% accuracy.

**Table 3:** **Foot strike identification**

**Participant**	**Primary acceleration**	**# Steps from video**	**# Steps by algorithm**	**Accuracy**	**Time difference of foot strikes from video (s)**
1	Forward	716	716	100.0%	0.02 ± 0.026
2	Forward	695	695	100.0%	0.02 ± 0.012
3	Forward	650	646	99.4%	0.02 ± 0.052
4	Forward	716	716	100.0%	0.01 ± 0.007
5	Forward	650	650	100.0%	0.02 ± 0.011
6	Vertical	663	662	99.9%	0.03 ± 0.030
7	Forward	758	758	100.0%	0.01 ± 0.007
8	Forward	726	726	100.0%	0.01 ± 0.006
9	Forward	752	752	100.0%	0.01 ± 0.011
10	Forward	635	635	100.0%	0.01 ± 0.009
11	Forward	647	647	100.0%	0.01 ± 0.012
12	Forward	607	607	100.0%	0.01 ± 0.008
13	Forward	617	617	100.0%	0.01 ± 0.008
14	Forward	652	652	100.0%	0.01 ± 0.010
15	Forward	746	746	100.0%	0.02 ± 0.020

Time difference is the average and standard deviation across all steps.

A sample of additional information calculated from these outcomes is shown in Additional file 1: Table S1; including, average walking speed, cadence, asymmetry, and step length throughout the 6MWT.

## Discussion

The 6MWT app accomplished the objectives of appropriately instructing the participant to start and stop walking, providing the distance walked within an acceptable accuracy, and accurately identify foot strike times. This supports the use of sensor-equipped smartphones for this physical rehabilitation application.

The most important output from the 6MWT is the total distance walked, since this is the measure of exercise capacity. When calculating distance walked from accelerometer signals, previous studies have used algorithms that require additional measurements from the participant, such as height or leg length [12,26] . Other formulas required experimental walking data to determine mathematical constants before distance could be accurately calculated [22,25]. The 6MWT is performed on track of known length, which in our application allowed the calculation of total distance based on the detection of turns and the calculation of average step length from the number of steps per length. This removed the necessity of additional measurements or complex algorithms to estimate the distance within a clinically-acceptable range. Our algorithm resulted in more accurate distance calculation than previous algorithms, based on appropriate turn detection and the 6MWT constraints. Accurate foot strike timing is not needed for the distance calculation, which uses number of steps and walkway length, but accurate foot strike timing can be used to calculate stride parameters as outcome measures in rehabilitation.

The one case where calculated distance error was greater than 1 m occurred when the trial ended shortly after a turn (i.e., 6 minutes elapsed just after completing a turn). The algorithm determined that the person was still turning and did not detect the 2 steps out of the turn, which covered 2.1 m. Physiotherapists consulted for this study indicated that a uncertainty of 1-2 m in a 6MWT is not clinically significant. The clinical minimal detectable change for the 6MWT varies by population, and ranges from 34 m [30] to 82 m [31].

Video verification revealed that participants usually took an extra step or two after data collection had stopped, due to their delayed response to the tone signaling the end of the trial. Using a smartphone for timing and to signal the start and end of the 6 minutes removes some variability from the traditional method of using a stopwatch and telling the person to stop when 6 minutes is reached; however, therapists also compensate for this variability by following the person and dropping a marker on the floor when 6 minutes are completed. An advantage of the smartphone approach would be for 6MWT testing in the community or in other cases where a therapist is not available to ensure appropriate end-of-test distance identification.

Our study showed that a peak detection algorithm that implements locking periods and compares the similarity of each peak to previous steps, rather than to a threshold, can produce accurate foot strike detection with a variable 50Hz sampling rate on a smartphone. Turns were identified by pelvis rotation. Steps at the beginning or end of a walkway, where the person’s pelvis was turned, were not counted or used to calculate gait characteristics. Thus, steps were counted when the trunk faced forward and the participant walked in their normal manner. The gold-standard video was recorded at approximately 30 frames per second. Since the real foot strike could have occurred one frame before or after the closest frame captured by the camera, a tolerance of 2 frames (0.07 seconds) was allowed for assessing foot strike accuracy. Of the 10225 foot strikes identified by the algorithm for all 15 participants, only 35 were not identified within this 0.07 s tolerance (99.66% accuracy), and 30 of those were from participant 6. These 35 steps were all identified within 0.3 s of the gold standard. All but one participant had average foot strike time differences less than 0.02 s, which were less than the gold-standard comparator tolerance.

Participant 6 was the only participant where the algorithm selected vertical acceleration as the primary signal for step detection. When the forward accelerometer signal does not contain identifiable foot strike peaks due to multiple peaks related to an atypical walking pattern, the vertical acceleration signal often provides viable peaks for step detection. To help understand these signal effects, the algorithm was re-run for participant 6 using the forward acceleration as the primary signal for comparison. Fluctuations in the forward acceleration signal caused 17 false step identifications, thus, the algorithm appropriately selected vertical acceleration. The maximum time difference between video and algorithm foot strike events was 0.3 seconds, occurring at the first step out of a turn. The actual foot strike event occurred while the person’s pelvis was still rotating (i.e., during a turn) so this step would not have been included if forward acceleration was used to detect steps. Since the vertical acceleration peak occurs after foot strike, the peak occurred after the turn and was detected using the vertical signal. The turn is not considered when identifying foot strike time, therefore the time was identified slightly after the actual foot strike, when the pelvis was no longer turning. Also, one step was not identified for participant 6. Future work could improve the results when the step detection algorithm selects the vertical acceleration signal. While the vertical signal peaks do not correspond with foot strike, they can be used to determine the time between two consecutive steps. In future work, this may be useful as a correction factor. When the forward signal was used to detect steps (i.e., default setting), the maximum time difference to the gold standard was 0.1 s.

During data collection for participant 3, the acceleration signals became disrupted and nearly flat for approximately 2.5 seconds, for unknown reasons. The algorithm identified this as a stop in the walkway and did not include this section when calculating the average step time and cadence. Thus, while 4 steps were not counted, calculation of gait characteristics and total distance walked was not negatively affected. This demonstrates that a smartphone may have some weaknesses when used as a sensor but the occurrence of a stop can easily be disproved or verified by the clinician running the test and the algorithm can still provide accurate gait information despite the disruption. Left and right steps were correctly identified for all participants when compared to video recordings.

While the algorithm was effective for the able-bodied test population, limitations include use with populations that do not have distinct foot strikes (i.e., shuffle gait with stroke or elderly) since steps would be missed, possibly resulting in an inaccurate distance estimate. Severe gait asymmetry could also adversely affect identification of left and right steps. Error could also be introduced for populations that cannot wear a belt that positions the phone appropriately throughout the test (i.e., belt slipping due to waist girth, etc.). The current algorithm only works with a straight walkway, although this is the typical method for executing the 6MWT [29].

## Conclusion

A novel algorithm was designed that uses a smartphone to run the 6MWT, accurately detect foot strike, and calculate the total distance walked during the test. The algorithm was validated on a sample of 15 able-bodied participants, generating superior distance calculation results compared with previous methods. This demonstrated that accurate foot strike and turn detection can be obtained using a smartphone platform, rather than a dedicated device, to provide clinically relevant outcome measures from the 6MWT. Clinicians can easily and affordably obtain the application for use in clinic and potentially have their patients administer their own 6MWT at home and send the results to the healthcare provider. The additional information derived from the 6MWT on gait symmetry, walking changes over time, and walking patterns could help with clinical decision-making.

Future work will validate the algorithm with patients receiving stroke or musculoskeletal rehabilitation, to assess the adaptive capabilities of the algorithm when used on a population with excessive gait asymmetry or irregular gait patterns.

## Additional file

Additional file 1: Table S1. Information derived from foot strike timing. Each row is one walkway length. Asymmetry is the difference between consecutive left and right step times, divided by the bilateral average.

## Abbreviations

6MWT: 6 minute walk test
SAM: StepWatch 3 activity monitor
COPD: Chronic obstructive pulmonary disease
App: Application
