# Supplementary material for: Novel algorithm for a smartphone-based 6-minute walk test application: algorithm, application development, and evaluation
Source: J Neuroeng Rehabil. 2015 Feb 20;12:19. doi: 10.1186/s12984-015-0013-9 (PMC4343050; doi:10.1186/s12984-015-0013-9)
Supplement: Additional file 1: Table S1. — Information derived from foot strike timing. Each row is one walkway length. Asymmetry is the difference between consecutive left and right step times, divided by the bilateral average. [file 12984_2015_13_MOESM1_ESM.docx]

# Additional file 1

Table S1: Information derived from foot strike timing. Each row is one walkway length. Asymmetry is the difference between consecutive left and right step times, divided by the bilateral average.

| Distance (m) | Number of steps | Average walking speed (m/s) | Average step length (m) | Average cadence (steps/min) | Asymmetry |
| --- | --- | --- | --- | --- | --- |
| 25.00 | 32 | 1.63 | 0.781 | 117.39 | 0.04 |
| 25.00 | 32 | 1.58 | 0.781 | 118.35 | 0.04 |
| 25.00 | 33 | 1.62 | 0.758 | 124.07 | 0.06 |
| 25.00 | 32 | 1.60 | 0.781 | 118.67 | 0.03 |
| 25.00 | 32 | 1.61 | 0.781 | 118.18 | 0.05 |
| 25.00 | 33 | 1.56 | 0.758 | 119.42 | 0.05 |
| 25.00 | 32 | 1.63 | 0.781 | 117.83 | 0.04 |
| 25.00 | 33 | 1.52 | 0.758 | 119.59 | 0.05 |
| 25.00 | 32 | 1.61 | 0.781 | 118.08 | 0.05 |
| 25.00 | 33 | 1.56 | 0.758 | 121.47 | 0.04 |
| 25.00 | 32 | 1.64 | 0.781 | 121.26 | 0.08 |
| 25.00 | 32 | 1.58 | 0.781 | 117.28 | 0.03 |
| 25.00 | 32 | 1.60 | 0.781 | 117.05 | 0.03 |
| 25.00 | 32 | 1.59 | 0.781 | 117.45 | 0.05 |
| 25.00 | 32 | 1.60 | 0.781 | 116.98 | 0.03 |
| 25.00 | 32 | 1.62 | 0.781 | 118.70 | 0.05 |
| 25.00 | 32 | 1.57 | 0.781 | 115.10 | 0.10 |
| 25.00 | 32 | 1.60 | 0.781 | 116.63 | 0.04 |
| 25.00 | 32 | 1.57 | 0.781 | 116.98 | 0.05 |
| 25.00 | 33 | 1.58 | 0.76 | 119.82 | 0.03 |
| 25.00 | 32 | 1.58 | 0.78 | 116.34 | 0.05 |
| 25.00 | 33 | 1.56 | 0.76 | 119.26 | 0.06 |
| 4.55 | 6 | 1.35 | 0.76 | 107.20 | 0.04 |
